# A radiomics nomogram for invasiveness prediction in lung adenocarcinoma manifesting as part-solid nodules with solid components smaller than 6 mm

**DOI:** 10.3389/fonc.2022.900049

**Published:** 2022-08-11

**Authors:** Teng Zhang, Chengxiu Zhang, Yan Zhong, Yingli Sun, Haijie Wang, Hai Li, Guang Yang, Quan Zhu, Mei Yuan

**Affiliations:** ^1^ Department of Radiology, The First Affiliated Hospital of Nanjing Medical University, Nanjing, China; ^2^ Shanghai Key Laboratory of Magnetic Resonance, East China Normal University, Shanghai, China; ^3^ Department of Radiology, Huadong Hospital Affiliated to Fudan University, Shanghai, China; ^4^ Department of Pathology, The First Affiliated Hospital of Nanjing Medical University, Nanjing, China; ^5^ Department of Thoracic Surgery, The First Affiliated Hospital of Nanjing Medical University, Nanjing, China

**Keywords:** radiomics, nomogram, adenocarcinoma of lung, neoplasm invasiveness, tomography, X-ray computed

## Abstract

**Objective:**

To investigate whether radiomics can help radiologists and thoracic surgeons accurately predict invasive adenocarcinoma (IAC) manifesting as part-solid nodules (PSNs) with solid components <6 mm and provide a basis for rational clinical decision-making.

**Materials and Methods:**

In total, 1,210 patients (mean age ± standard deviation: 54.28 ± 11.38 years, 374 men and 836 women) from our hospital and another hospital with 1,248 PSNs pathologically diagnosed with adenocarcinoma *in situ* (AIS), minimally invasive adenocarcinoma (MIA), or IAC were enrolled in this study. Among them, 1,050 cases from our hospital were randomly divided into a derivation set (n = 735) and an internal validation set (n = 315), 198 cases from another hospital were used for external validation. Each labeled nodule was segmented, and 105 radiomics features were extracted. Least absolute shrinkage and selection operator (LASSO) was used to calculate Rad-score and build the radiomics model. Multivariable logistic regression was conducted to identify the clinicoradiological predictors and establish the clinical-radiographic model. The combined model and predictive nomogram were developed based on identified clinicoradiological independent predictors and Rad-score using multivariable logistic regression analysis. The predictive performances of the three models were compared *via* receiver operating characteristic (ROC) curve analysis. Decision curve analysis (DCA) was performed on both the internal and external validation sets to evaluate the clinical utility of the nomogram.

**Results:**

The radiomics model showed superior predictive performance than the clinical-radiographic model in both internal and external validation sets (Az values, 0.884 vs. 0.810, *p* = 0.001; 0.924 vs. 0.855, *p* < 0.001, respectively). The combined model showed comparable predictive performance to the radiomics model (Az values, 0.887 vs. 0.884, *p* = 0.398; 0.917 vs. 0.924, *p* = 0.271, respectively). The clinical application value of the nomogram developed based on the Rad-score, maximum diameter, and lesion shape was confirmed, and DCA demonstrated that application of the Rad-score would be beneficial for radiologists predicting invasive lesions.

**Conclusions:**

Radiomics has the potential as an independent diagnostic tool to predict the invasiveness of PSNs with solid components <6 mm.

## Introduction

With the increasing use of low-dose computed tomography (LDCT) in the screening of high-risk populations for lung cancer, the detection rate of part-solid nodules (PSNs) has been increasing, especially in Asian women and non-smokers (1–4). Previous research has shown that persistent PSNs are highly correlated with early-stage lung adenocarcinoma, including invasive adenocarcinoma (IAC), minimally invasive adenocarcinoma (MIA), and adenocarcinoma *in situ* (AIS) (5–7).

Accurate differentiation between IAC and AIS/MIA appearing as PSNs is critical and can determine the patient’s treatment options. Unlike IAC, AIS/MIA can be resected by limited wedge resection or segmentectomy rather than lobectomy to maximize the preservation of functional pulmonary parenchyma. Moreover, lymph node exploration is not required for AIS/MIA (8, 9). For clinical management, as long as the lesions suspected of AIS/MIA remain stable, the strategy of conservative periodic follow-up with CT, with surgical resection in case of lesion growth, has been widely accepted by clinicians, ultimately avoiding unnecessary surgery for patients (4, 10, 11).

One of the key factors to predict the invasiveness of PSNs is the assessment of the size of the solid component within the nodules, which is highly correlated with the pathologically invasive foci of adenocarcinomas (12–14). For PSNs, a size criterion of solid component diameter ≥6 mm is widely accepted to discriminate IAC from AIS/MIA on CT, which is also the newly revised threshold standard for T-factor staging of adenocarcinoma. Most IACs commonly manifest as PSNs with solid components ≥6 mm and can be easily and accurately diagnosed by radiologists and thoracic surgeons (4, 15, 16). A statement from the Fleischner Society suggested that surgical resection should be considered if solid components were ≥6 mm in PSNs, while yearly surveillance CT is recommended for PSNs with solid components <6 mm (4, 17). However, due to the insufficient CT resolution, the ground-glass components of PSNs may contain invasive foci that cannot be recognized by the naked eyes; a large number of IAC cases also present as PSNs with solid component <6 mm or even non-solid nodules (NSNs), which were difficult to distinguish from AIS/MIA, and closer follow-up is needed for these lesions (18). Ahn et al. (19) indicated that the sizes of the solid component measured on CT images commonly underestimate the real size of invasive foci on pathology. Therefore, it is a great challenge for radiologists to predict the invasiveness of PSNs with a solid component <6 mm and NSNs because of their pathological diversity.

Many studies regarding the invasiveness prediction of PSNs have been reported with different methods, including radiographic feature evaluation and quantitative analysis (20–22). However, these studies have limitations: 1) there are no limits set on the size of solid components in PSNs. If too many PSNs with a solid component ≥6 mm (the pathological type is mostly IAC) are included in a study, the predictive performance may be exaggerated; 2) many evaluated radiographic features show an overlap between IAC and AIS/MIA; 3) previous studies used varied quantitative parameters and reported different results, making it unclear whether these studies can help radiologists improve the prediction performance.

Radiomics has been widely used to establish diagnosis and prediction models for tumor grading and staging, treatment outcome evaluation, and prognosis prediction by extracting and selecting predefined subtle image features (23, 24). Sun et al. (25) and Yuan et al. (26) have successfully used radiomics to predict the invasiveness of NSNs and PSNs, respectively. However, few studies have focused on the prediction of invasiveness of PSNs with a solid component <6 mm, whose diagnosis remains challenging for radiologists. Therefore, the purpose of our study was to investigate whether radiomics is able to help radiologists accurately predict IAC manifesting as PSNs with solid components <6 mm and provide a basis for rational clinical decision-making.

## Materials and methods

The ethical committee of our hospital approved this retrospective study and waived the informed consent for the patients (approval number: 2021-SR-053).

### Data source and patient selection

We searched the institution’s database and collected the clinical and imaging data of 3,326 patients who underwent surgery in our hospital and were pathologically diagnosed with AIS, MIA, or IAC from January 2015 to December 2020. The dataset of our hospital included 1,012 patients (mean age ± standard deviation, 54.16 ± 11.21 years, 316 men and 696 women) with 1,050 PSNs satisfying the following inclusion criteria: 1) the lesions manifested as PSNs on CT imaging, with the maximum diameter of the solid components <6 mm (excluding bronchi and vessels); 2) the maximum diameter of the PSNs was 5–30 mm; 3) non-enhanced CT scans were performed within 2 weeks prior to surgery; 4) lesions were completely removed, and the pathological diagnosis was unambiguous. In accordance with previous studies, the CT threshold of the solid components in PSNs was set to >-188 HU (27). Three-dimensional (3D) Slicer software (version 4.12; National Institutes of Health; https://www.slicer.org) can automatically identify solid components in PSNs. According to the automatic segment results of the solid components in PSNs in 3D Slicer software, authors #1 and #3 (with 10 and 7 years’ experience in chest CT imaging, respectively) separately measured the maximum diameter of the solid components from the axial, coronal, and sagittal images in Picture Archiving and Communication System (PACS) (**Supplementary Figure S1**). PSNs with solid components greater than 6 mm measured from any direction will be excluded from the study. Author #9, a radiologist with 16 years’ experience, reviewed the measurement results of authors #1 and #3 to reach a consensus.

Finally, the dataset from our hospital was randomly divided into a derivation set (735 cases: 67 AIS, 316 MIA, and 352 IAC) and an internal validation set (315 cases: 29 AIS, 135 MIA, and 151 IAC) at a ratio of 7:3.

**Figure 1:**
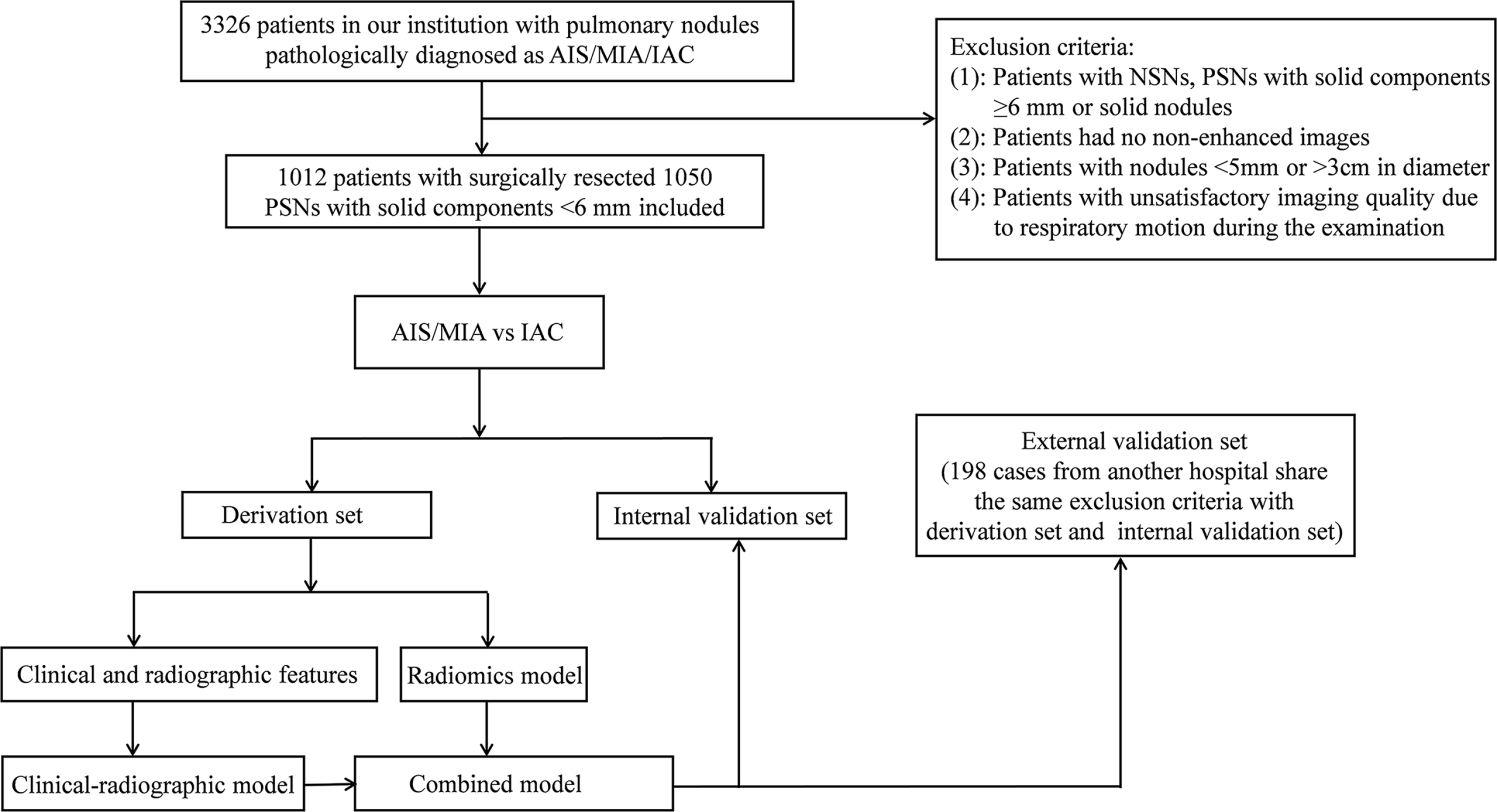
The workflow of our study.

Authors #1, #3, and #9 used the same methods andreviewed patients who underwent surgery in another hospital and were pathologically diagnosed with AIS, MIA, or IAC from January 2020 to December 2020. Finally, 198 cases (25 AIS, 79 MIA, and 94 IAC) were enrolled in our study as the external validation set. The workflow was illustrated in **Figure 1**.

None of the 597 IAC cases included in our study had lymph node metastases. Only 16 IAC cases were classified as pT2 stage because of the presence of pleural invasion and the rest were all classified as pT1 stage according to the eighth edition of the tumor, node, and metastasis (TNM) classification of lung cancer.

### Diagnostic criteria

The resection specimens were fixed with formalin, embedded in paraffin, sectioned, and stained with hematoxylin and eosin. Author #6, a pathologist with 26 years’ work experience, reviewed the pathological classification of all specimens based on the 2011 classification criteria for lung adenocarcinoma proposed by International Association for the Study of Lung Cancer/American Thoracic Society/European Respiratory Society (IASLC/ATS/ERS).

### CT examination methods

All patients underwent CT examination using one of the four CT scanners: SOMATOM Definition AS+, SOMATOM Sensation 16, and GE Discovery CT750 HD of our hospital and GE LightSpeed VCT of another hospital. The detailed scan and reconstruction parameters and the number of patients performed by each scanner were shown in **Supplementary Table S1**.

### Establishing the clinical-radiographic model

The patient’s clinical information was collected from electronic medical records, including: 1) age, 2) sex, 3) smoking history, 4) carcinoembryonic antigen (CEA) level, 5) history of chronic obstructive pulmonary disease (COPD), 6) history of other cancers, and 7) family history of lung cancer. Authors #1 and #3, who did not know the patients’ pathological diagnosis, interpreted the CT images individually under lung window settings (width, 1,200 Hu; level, -600 Hu). The radiographic features of all lesions evaluated in this study include the following: 1) lesion location; 2) maximal axial diameter; 3) maximum axial diameter of the solid component; 4) consolidation-to-tumor ratio (CTR); 5) border (undefined or defined); 6) shape (round/oval or irregular); 7) vacuole sign (lesions with cystic cavities with the diameter <5 mm); 8) air bronchogram sign (dilated bronchioles observed in lesions); 9) microvascular sign (lesions with convergent, dilated, or tortuous supplying vessels); and 10) pleural indentation sign (pleura adjacent to lesions showed thickening or contraction). The CTR (%) was calculated as 100×(maximum axial diameter of the solid component/maximum axial diameter of the lesion) referring to previous studies (28, 29). Kappa values and intraclass correlation coefficients (ICCs) were calculated to assess the consistency of the two authors’ evaluations. To reach a consensus, author #9 rechecked the image interpretation results.

### Nodule segmentation and radiomics feature extraction

CT images (DICOM format) were retrieved from the institution archive and loaded into a personal computer for further analysis. The volume of interest (VOI) was automatically segmented using a homemade software MultiLabel (version 1.1, Shanghai Key Laboratory of Magnetic Resonance, East China Normal University, China). Manual adjustment for the precise edge of the VOI was performed by author #1 if the border of the lesion was undefined or to ensure the large vessels and bronchioles were excluded from the VOI (**Supplementary Figure S2**). Author #9 reviewed the VOIs to ensure accurate segmentation. Then, the whole set of CT images with VOI segmentation information was converted to NII format for further radiomics analysis.

We normalized all images with the following formula: *f*(*x*)=1000∗(*x*−*μ*_*x*_)/*σ*_*x*_, where *μ_x_* and *σ_x_* denote the mean and standard deviation of the image intensity, respectively, before 3D radiomics features were extracted from the original image with PyRadiomics (Ver. 3.0) (30). We extracted 105 commonly used features in radiomics analysis, including 18 gray-level histogram features (e.g., mean, kurtosis, skewness), 14 shape features (e.g., compactness, sphericity), and 73 high-order texture features [gray-level co-occurrence matrix (GLCM), gray-level run-length matrix (GLRLM), gray-level size-zone matrix (GLSZM), and neighborhood gray-tone difference matrix (NGTDM)]. Most features defined by Pyradiomics follow feature definitions as described by the Imaging Biomarker Standardization Initiative (IBSI) (31).

In order to check the inter- and intra-observer reproducibility of the 105 radiomics features that we extracted, 60 cases (20 AIS, 20 MIA, and 20 IAC) were randomly chosen for analysis. Author #1 and author #3 repeated the nodule segmentation procedure for the selected 60 cases separately approximately 3 months later. The intra- and inter-observer agreement of the 105 extracted features were assessed by interclass correlation coefficients and ICCs. An ICC greater than 0.75 indicated good reproducibility of the feature extraction.

### Feature selection and rad-score building

Firstly, all features were normalized with z-score (subtracted mean value and divided by standard deviation), then minimum-Redundancy Maximum Relevance (mRMR) was used to remove redundant features. The remaining 30 features were used to build the radiomics model with least absolute shrinkage and selection operator (LASSO), a classifier suitable for high-dimensional data regression. Parameter and thus the appropriate number of the most weighted predictive features was determined using a 10-fold cross-validation over the derivation set. To minimize the number of features in the final model, we chose the model with the least number of features and a binomial deviance within 1 standard deviation from the minimum binomial deviance.

After the model had been built, Rad-score, namely, the predictive probability of the radiomics model for each patient, was calculated *via* a linear combination of the selected most weighted features with their respective coefficients.

### Statistical analyses

Univariable and multivariable logistic regression analyses were carried out on the clinical and radiographic features of the derivation set to determine the independent predictors for IAC and establish the clinical-radiographic model. The independent predictors and Rad-score were analyzed using multivariable logistic regression; thus, a combined model and an individual prediction nomogram were constructed. Receiver operating characteristic (ROC) curve analysis was used to evaluate the performance of the clinical-radiographic model, radiomics model, and combined model in the derivation, internal validation, and external validation sets. The optimal cutoff value was determined by Youden index in the ROC analysis. DeLong’s test was also used to compare the performance of the models. Model evaluation metrics such as positive predictive value (PPV), negative predictive value (NPV), F1-score, and Matthews correlation coefficient (MCC) were calculated to identify the best prediction model. Waterfall plot was used to show the prediction probability of all patients, and calibration curves were plotted to analyze the diagnostic performance of the nomogram in each dataset. Hosmer–Lemeshow test and decision curve analysis (DCA) were used to evaluate the goodness of fit and clinical value of the nomogram.

Statistical analysis was performed using IBM SPSS software (version 26.0; https://www.ibm.com) and R software (version 4.1.0; https://www.r-project.org). Specifically, we used rms package for calibration analysis, ResourceSelection package for Hosmer–Lemeshow test, rmda for DCA, mRMRe for feature selection, and glmnet for LASSO. *p-*values <0.05 were considered statistically significant.

## Results

### Predictive performance of the clinical-radiographic model

**Supplementary Table S2** showed the inter-observer agreement for the radiographic sign evaluation and measurement of PSNs. The Kappa values for lesion measurement and radiographic features evaluation were medium to high.

It can be seen from **Table 1** that there were no statistically significant differences between the derivation set and internal/external validation set in the comparison of clinical and radiographic features.

**Table 1:** Comparison of clinical and radiographic characteristics between derivation and internal/external validation sets.

Characteristics	Derivation set (n = 735)	Internal validation set (n = 315)	External validation set (n = 198)	*p* value	*p’* value
**Clinical Characteristics**					
Age (years)	54.27 ± 10.90	53.91 ± 11.91	54.90 ± 12.29	0.364^a^	0.512^a^
Sex (Men/Women)	227 (30.9)/508 (69.1)	101 (32.1)/214 (67.9)	58 (29.3)/140 (70.7)	0.760^b^	0.666^b^
Smoking history (Yes/No)	89 (12.1)/646 (87.9)	36 (11.4)/279 (88.6)	20 (10.1)/178 (89.9)	0.835^b^	0.435^b^
CEA (ng/ml)	1.96 ± 1.28	2.08 ± 1.60	2.02 ± 1.27	0.189^a^	0.568^a^
History of COPD (Yes/No)	50 (6.8)/685 (93.2)	18 (5.7)/297 (94.3)	9 (4.5)/189 (95.5)	0.603^b^	0.247^b^
History of other cancers (Yes/No)	53 (7.2)/682 (92.8)	21 (6.7)/294 (93.3)	16 (8.1)/182 (91.9)	0.854^b^	0.678^b^
Family history of lung cancer (Yes/No)	34 (4.6)/701 (95.4)	15 (4.8)/300 (95.2)	14 (7.1)/184 (92.9)	0.676^b^	0.167^b^
**Radiographic Characteristics**					
Lesion location				0.833^b^	0.224^b^
Right upper lobe	271 (36.9)	117 (37.1)	61 (30.8)		
Right middle lobe	50 (6.8)	22 (7.0)	17 (8.6)		
Right lower lobe	119 (16.2)	49 (15.6)	30 (15.2)		
Left upper lobe	182 (24.7)	86 (27.3)	63 (31.8)		
Left lower lobe	113 (15.4)	41 (13.0)	27 (13.6)		
Maximum diameter (cm)	1.25 ± 0.43	1.26 ± 0.43	1.20 ± 0.41	0.236^a^	0.215^a^
Maximum diameter of the solid component (cm)	0.31 ± 0.13	0.30 ± 0.13	0.30 ± 0.14	0.493^a^	0.738^a^
CTR (%)	25.89 ± 11.23	26.09 ± 13.95	27.12 ± 13.01	0.359^a^	0.474^a^
Lesion shape				0.717^b^	0.175^b^
Round/Oval	537 (73.1)	226 (71.7)	135 (68.2)		
Irregular	198 (26.9)	89 (28.3)	63 (31.8)		
Lesion border				0.920^b^	0.350^b^
Defined	587 (79.9)	250 (79.4)	164 (82.8)		
Undefined	148 (20.1)	65 (20.6)	34 (17.2)		
Vacuole sign (Yes/No)	79 (10.7)/656 (89.3)	32 (10.2)/283 (89.8)	17 (8.6)/181 (91.4)	0.861^b^	0.374^b^
Air bronchogram (Yes/No)	81 (11.0)/654 (89.0)	28 (8.9)/287 (91.1)	14 (7.1)/184 (92.9)	0.354^b^	0.103^b^
Microvascular sign (Yes/No)	226 (30.7)/509 (69.3)	98 (31.1)/217 (68.9)	63 (31.8)/135 (68.2)	0.850^b^	0.773^b^
Pleural indentation (Yes/No)	228 (31.0)/507 (69.0)	110 (34.9)/205 (65.1)	73 (36.9)/125 (63.1)	0.243^b^	0.118^b^

CEA, carcinoembryonic antigen; COPD, chronic obstructive pulmonary disease; CTR, consolidation-to-tumor ratio.

Values are presented as no. (%) or mean ± standard deviation.

^a^Mann–Whitney U test. ^b^Pearson’s chi-square test and Fisher’s exact test.

p value: Derivation set vs. Internal validation set; p’ value: Derivation set vs. External validation set.

**Figure 2:**
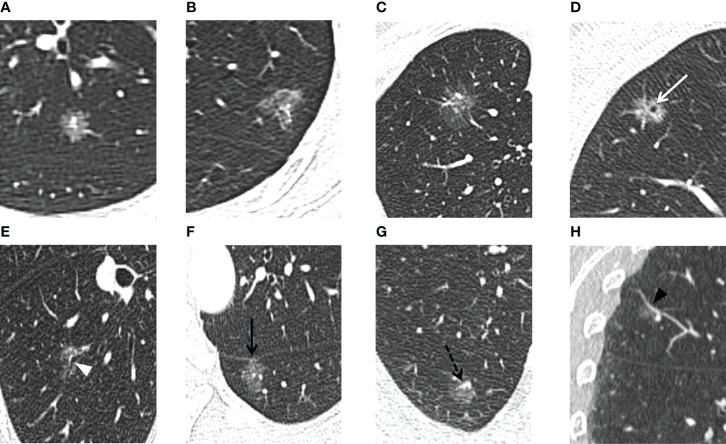
For the PSNs included in our study, thin-section CT radiographic features were evaluated. The lesion shape is evaluated as **(A)** round/oval or **(B)** irregular. The lesion border is evaluated as **(A, B)** defined and **(C)** undefined. **(D)** Vacuole sign: MIA in the right upper lobe exhibits a bubble-like lucency within the nodule (white arrow). **(E)** Air bronchogram sign: MIA in the right lower lobe shows air-filled bronchi present inside the lesion (white arrowhead). **(F)** Pleural indentation: MIA in the left lower lobe shows pleural indentation adjacent to the oblique fissure (black arrow). **(G, H)** Microvascular sign: IAC in the right upper lobe shows a small adjacent pulmonary vessel entering the lesion (black dotted arrow). Reverse tracing shows that the blood vessel is a branch of the pulmonary artery. Coronal reconstruction better shows the dilation of the supplying vessel (black arrowhead).

**Table 2** and **Figure 2** present the comparison on the clinical and radiographic features between AIS/MIA and IAC in the derivation, internal validation, and external validation sets. Univariable and multivariable logistic regression analyses in the derivation set revealed the maximum diameter [odds ratio (OR) 3.62, 95% confidence interval (CI) 2.26–5.80, *p <* 0.001], lesion shape (OR 1.89, 95% CI 1.26–2.81, *p* = 0.002), vacuole sign (OR 1.89, 95% CI 1.07–3.32, *p* = 0.028), microvascular sign (OR 1.91, 95% CI 1.31–2.79, *p* = 0.001), and maximum diameter of the solid component (OR 26.83, 95% CI 6.81–105.76, *p* < 0.001) were independent predictors for IAC.

**Table 2:** Comparison on the clinical and radiographic characteristics between AIS-MIA and IAC in the derivation, internal validation, and external validation sets.

Characteristics	Derivation set (n =735)		*p* value	Internal validation set (n = 315)		*p* value	External validation set (n = 198)		*p* value
	AIS-MIA group (n = 383)	IAC group (n = 352)		AIS-MIA group (n = 164)	IAC group (n = 151)		AIS-MIA group (n = 104)	IAC group (n = 94)	
**Clinical Characteristics**									
Age (years)	52.47 ± 11.18	56.23 ± 10.24	0.000^a^	52.31 ± 12.62	55.64 ± 10.88	0.016^a^	52.21 ± 12.89	58.99 ± 10.19	0.000^a^
Sex (Men/Women)	101 (26.4)/282 (73.6)	126 (35.8)/226 (64.2)	0.006^b^	51 (31.1)/113 (68.9)	50 (33.1)/101 (66.9)	0.702^b^	27 (26.0)/77 (74.0)	31 (33.0)/63 (67.0)	0.279^b^
Smoking history (Yes/No)	34 (8.9)/349 (91.1)	55 (15.6)/297 (84.4)	0.005^b^	11 (6.7)/153 (93.3)	25 (16.6)/126 (83.4)	0.006^b^	8 (7.7)/96 (92.3)	12 (12.8)/82 (87.2)	0.237^b^
CEA (ng/ml)	1.81 ± 1.14	2.12 ± 1.40	0.003^a^	1.97 ± 1.75	2.20 ± 1.41	0.017^a^	1.83 ± 1.04	2.23 ± 1.45	0.025^a^
History of COPD (Yes/No)	21 (5.5)/362 (94.5)	29 (8.2)/323 (91.8)	0.138^b^	6 (3.7)/158 (96.3)	12 (7.9)/139 (92.1)	0.101^b^	4 (3.8)/100 (96.2)	5 (5.3)/89 (94.7)	0.877^b^
History of other cancers (Yes/No)	20 (5.2)/363 (94.8)	33 (9.4)/319 (90.6)	0.030^b^	7 (4.3)/157 (95.7)	14 (9.3)/137 (90.7)	0.075^b^	7 (6.7)/97 (93.3)	9 (9.6)/85 (90.4)	0.463^b^
Family history of lung cancer (Yes/No)	20 (5.2)/363 (94.8)	14 (4.0)/338 (96.0)	0.422^b^	7 (4.3)/157 (95.7)	8 (5.3)/143 (94.7)	0.668^b^	6 (5.8)/98 (94.2)	8 (8.5)/86 (91.5)	0.452^b^
**Radiographic Characteristics**									
Lesion location			0.571^b^			0.217^b^			0.200^b^
Right upper lobe	148 (38.6)	123 (34.9)		64 (39.0)	53 (35.1)		27 (26.0)	34 (36.2)	
Right middle lobe	25 (6.5)	25 (7.1)		7 (4.3)	15 (9.9)		10 (9.6)	7 (7.4)	
Right lower lobe	58 (15.1)	61 (17.3)		28 (17.1)	21 (13.9)		21 (20.2)	9 (9.6)	
Left upper lobe	99 (25.9)	83 (23.6)		47 (28.7)	39 (25.8)		31 (29.8)	32 (34.0)	
Left lower lobe	53 (13.9)	60 (17.1)		18 (10.9)	23 (15.3)		15 (14.4)	12 (12.8)	
Maximum diameter (cm)	1.09 ± 0.38	1.42 ± 0.42	0.000^a^	1.08 ± 0.34	1.45 ± 0.44	0.000^a^	0.99 ± 0.26	1.42 ± 0.43	0.000^a^
Maximum diameter of the solid component (cm)	0.26 ± 0.11	0.35 ± 0.14	0.000^a^	0.25 ± 0.10	0.34 ± 0.13	0.000^a^	0.24 ± 0.10	0.37 ± 0.14	0.000^a^
CTR (%)	25.72 ± 11.53	26.07 ± 10.90	0.424^a^	25.67 ± 13.22	26.54 ± 14.74	0.793^a^	25.77 ± 12.79	28.62 ± 13.15	0.112^a^
Lesion shape			0.000^b^			0.000^b^			0.000^b^
Round/Oval	326 (85.1)	211 (59.9)		141 (86.0)	85 (56.3)		83 (79.8)	52 (55.3)	
Irregular	57 (14.9)	141 (40.1)		23 (14.0)	66 (43.7)		21 (20.2)	42 (44.7)	
Lesion border			0.190^b^			0.014^b^			0.118^b^
Defined	313 (81.7)	274 (77.8)		139 (84.8)	111 (73.5)		82 (78.8)	82 (87.2)	
Undefined	70 (18.3)	78 (22.2)		25 (15.2)	40 (26.5)		22 (21.2)	12 (12.8)	
Vacuole sign (Yes/No)	24 (6.3)/359 (93.7)	55 (15.6)/297 (84.4)	0.000^b^	16 (9.8)/148 (90.2)	16 (10.6)/135 (89.4)	0.805^b^	5 (4.8)/99 (95.2)	12 (12.8)/82 (87.2)	0.046^b^
Air bronchogram (Yes/No)	23 (6.0)/360 (94.0)	58 (16.5)/294 (83.5)	0.000^b^	7 (4.3)/157 (95.7)	21 (13.9)/130 (86.1)	0.003^b^	6 (5.8)/98 (94.2)	8 (8.5)/86 (91.5)	0.452^b^
Microvascular sign (Yes/No)	68 (17.8)/315 (82.2)	158 (44.9)/194 (55.1)	0.000^b^	30 (18.3)/134 (81.7)	68 (45.0)/83 (55.0)	0.000^b^	25 (24.0)/79 (76.0)	38 (40.4)/56 (59.6)	0.013^b^
Pleural indentation (Yes/No)	95 (24.8)/288 (75.2)	133 (37.8)/219 (62.2)	0.000^b^	42 (25.6)/122 (74.4)	68 (45.0)/83 (55.0)	0.000^b^	30 (28.8)/74 (71.2)	43 (45.7)/51 (54.3)	0.014^b^

AIS, adenocarcinoma in situ; MIA, minimally invasive adenocarcinoma; IAC, invasive adenocarcinoma; CEA, carcinoembryonic antigen; COPD, chronic obstructive pulmonary disease; CTR, consolidation-to-tumor ratio.

Values are presented as no. (%) or mean ± standard deviation.

^a^Mann–Whitney U test. ^b^Pearson’s chi-square test and Fisher’s exact test.

**Figure 3:**
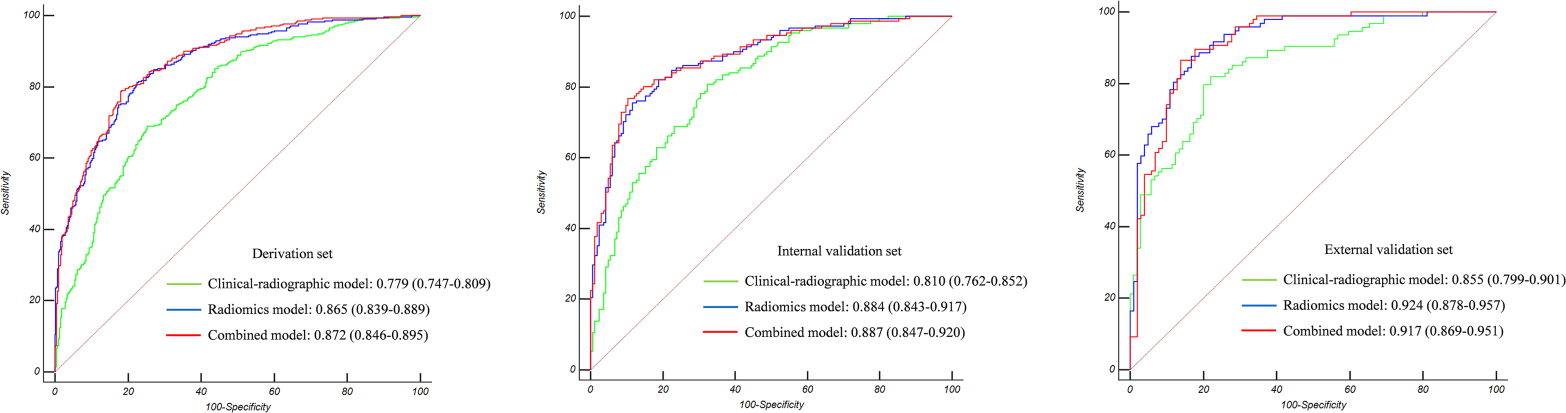
The Az value of the three models in the derivation set, internal validation set, and external validation set. Radiomics showed superior predictive performance than clinical-radiographic models in all sets; however, the combined models showed comparable predictive performance to the radiomics models.

The Az value of the clinical-radiographic model was 0.779 (95% CI 0.747–0.809) in the derivation set, 0.810 (95% CI 0.762–0.852) in the internal validation set, and 0.855 (95% CI 0.799–0.901) in the external validation set (**Figure 3**).

### Feature selection and rad-score building

The inter-observer ICCs, calculated on the basis of author #1’s first-extracted 105 features and those of author #3 ranged from 0.80 to 0.99 (**Supplementary Table S3**). The intra-observer ICCs, calculated based on author #1’s twice feature extraction ranged from 0.84 to 0.99 (**Supplementary Table S4**). Therefore, the 105 features we extracted proved robust and achieved satisfactory inter- and intra-observer reproducibility.

**Figure 4:**
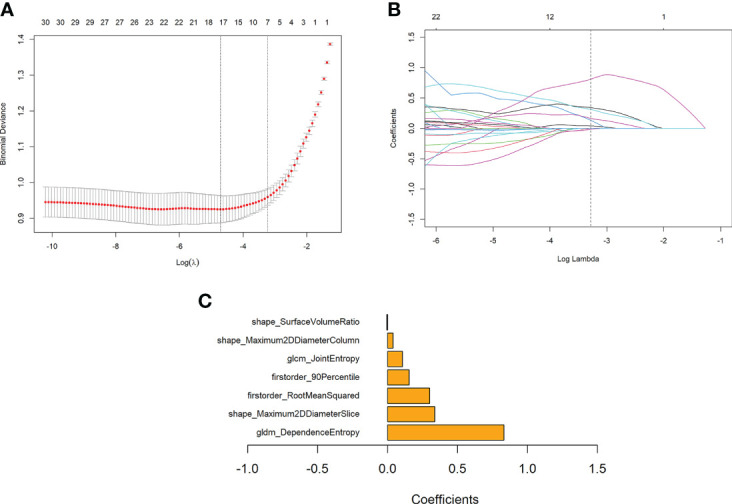
Features selection using the LASSO regression model and the selection of the tuning parameter λ. **(A)** Change of binomial deviance with log(λ). The maximum log(λ) corresponding to the binomial deviance within 1 standard error from the minimum binomial deviance was chosen for the final model. **(B)** Change of the number of features with non-zero coefficients with log(λ), as determined in a 10-fold validation. **(C)** Coefficients of the seven features retained in the model.

A Rad-score was calculated for each patient based on seven features with non-zero coefficients selected from the 105 robust radiomics features using a LASSO logistic regression model (λ = 0.039727) (**Figures 4A, B** **)**.

Rad-score = 0.831176 * gldm_DependenceEntropy

+ 0.301168 * firstorder_RootMeanSquared

- 0.004266 * shape_SurfaceVolumeRatio

+ 0.336894 * shape_Maximum2DDiameterSlice

+ 0.155065 * firstorder_90Percentile

+ 0.107055 * glcm_JointEntropy

+ 0.039099 * shape_Maximum2DDiameterColumn - 0.101629

The bar chart of the coefficients of features used in the model is shown in **Figure 4C**.

The Az value of the radiomics model was 0.865 (95% CI 0.839–0.889) in the derivation set, 0.884 (95% CI 0.843–0.917) in the internal validation set, and 0.924 (95% CI 0.878–0.957) in the external validation set (**Figure 3**).

### Prediction nomogram construction and validation

**Figure 5:**
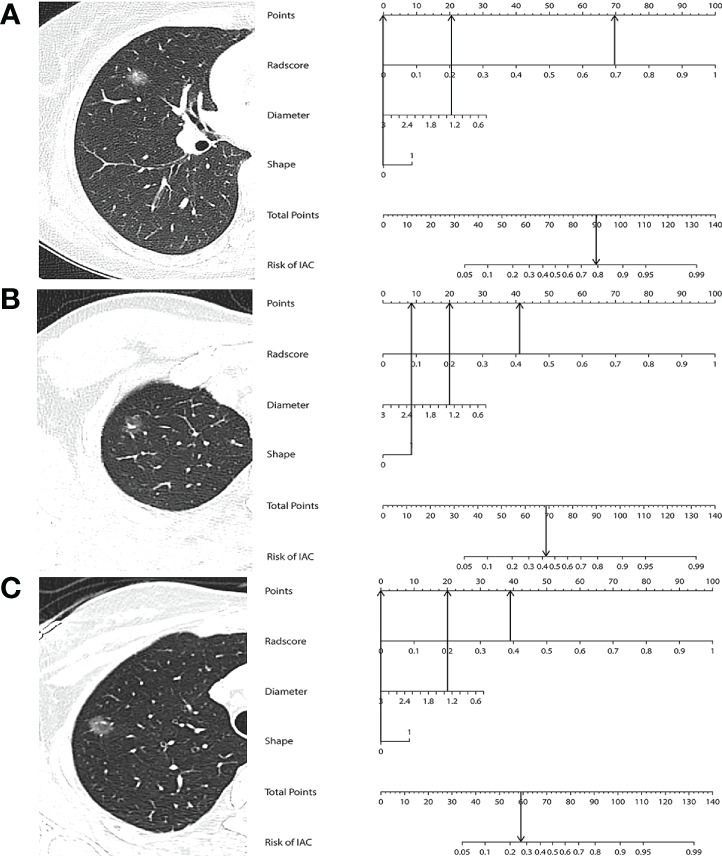
**(A)** A 45-year-old woman with IAC in the right middle lobe shows a regular shape and with a diameter of 1.26 cm. The nomogram shows that this case had a total of 90 points after summing all points (69 + 21 + 0), which corresponds to a 79.6% probability of IAC. **(B)** A 26-year-old woman with MIA in the right upper lobe shows an irregular shape and with a diameter of 1.32 cm. The nomogram shows that this case had a total of 69 points after summing all points (41 + 20 + 8), which corresponds to a 44.3% probability of IAC. **(C)** A 36-year-old woman with AIS in the right upper lobe shows a regular shape and with a diameter of 1.34 cm. The nomogram shows that this case had a total of 59 points after summing all points (39 + 20 + 0), which corresponds to a 25.9% probability of IAC.

Multivariable logistic regression analysis identified the Rad-score (OR 2,232.55, 95% CI 650.95–7656.89, *p* < 0.001), maximum diameter (OR 0.40, 95% CI 0.21–0.75, *p* = 0.004), and lesion shape (OR 1.94, 95% CI 1.23–3.06, *p* = 0.004) as independent predictors for IAC. All of these parameters were used to develop a prediction nomogram. Representative examples of the nomogram to predict the invasiveness are given in **Figures 5**.

The Az value of the combined model was 0.872 (95% CI 0.846–0.895) in the derivation set, 0.887 (95% CI 0.847–0.920) in the internal validation set, and 0.917 (95% CI 0.869–0.951) in the external validation set (**Figure 3**). It can be seen from the detailed metrics listed in **Table 3** that the radiomics models showed superior predictive performance than the clinical-radiographic models in all sets; however, the combined models showed comparable predictive performance to the radiomics models.

**Table 3:** Effectiveness of the three models in discriminating AIS-MIA from IAC in the derivation, internal validation, and external validation sets.

	Az (95% CI)		Cutoff value	SEN (%)	SPE (%)	PPV (%)	NPV (%)	ACC (%)	F1-score	MCC	Model-fitting information	
											AIC (%)	R^2^ value
**Derivation set**												
Clinical-radiographic model	0.779 (0.747-0.809)		>0.471	64.8	76.8	71.9	70.3	71.0	0.682	0.443	47.5	0.225
Radiomics model	0.865 (0.839-0.889)		>0.456	75.3	81.5	78.9	78.2	78.5	0.770	0.569	63.2	0.398
Combined model	0.872 (0.846-0.895)		>0.494	78.7	82.0	80.1	80.7	80.4	0.794	0.607	64.5	0.415
**Internal validation set**												
Clinical-radiographic model	0.810 (0.762-0.852)		>0.416	66.9	76.8	72.7	71.6	72.1	0.697	0.439	53.0	0.279
Radiomics model	0.884 (0.843-0.917)		>0.531	78.1	83.5	81.4	80.6	81.0	0.797	0.618	66.8	0.444
Combined model	0.887 (0.847-0.920)		>0.560	80.1	84.8	82.9	82.2	82.5	0.815	0.650	68.4	0.466
**External validation set**												
Clinical-radiographic model	0.855 (0.799-0.901)		>0.419	71.3	80.8	77.0	75.7	76.3	0.740	0.523	60.9	0.367
Radiomics model	0.924 (0.878-0.957)		>0.444	83.5	84.2	83.5	84.2	83.8	0.835	0.613	73.3	0.535
Combined model	0.917 (0.869-0.951)		>0.472	84.5	86.1	85.4	85.3	85.4	0.850	0.707	73.8	0.542

Az, area under the receiver operating curve; CI, confidence interval; SEN, sensitivity; SPE, specificity; PPV, positive predictive value; NPV, negative predictive value; ACC, accuracy; MCC, Matthews correlation coefficient; AIC, Akaike information criterion; AIS, adenocarcinoma in situ; MIA, minimally invasive adenocarcinoma; IAC, invasive adenocarcinoma.

**Figure 6:**
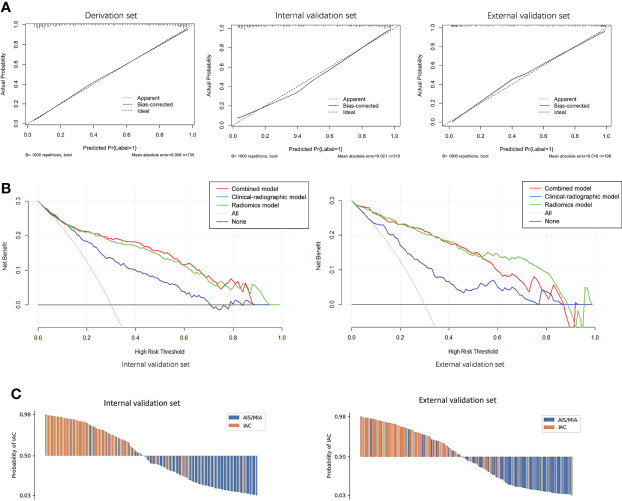
**(A)** Calibration curves of the combined model indicated good agreement between the predicted probability and actual occurrence in the derivation, internal validation, and external validation sets. **(B)** Decision curve analysis on both the internal and external validation sets for the models with and without Rad-score. It can be seen that if the threshold probability of a patient is in the range of 0.10~0.85, using a model with the Rad-score to predict the invasive lesion would be more beneficial than using one without the Rad-score. **(C)** Waterfall plot of the combined model showing the predicted probabilities of internal and external validation sets. It can be seen clearly that the combined model can differentiate IAC from AIS/MIA well.

The calibration curve of the radiomics nomogram smoothed with bootstrapping also indicated good agreement between predicted probability and actual occurrence in the derivation, internal validation, and external validation sets (**Figure 6A**). The Hosmer–Lemeshow test indicated no significant difference between the combined model’s predictions and the observed values in the derivation, internal validation, and external validation sets (*p* = 0.779, *p* = 0.580, *p* = 0.209, respectively), implying the model’s good generalization.

**Figure 6B** showed the decision curves of the developed models on both the internal and external validation sets. It would be more beneficial to use a model with Rad-score for identifying the invasive lesions than that without the Rad-score if the threshold probability of a patient was in the range of 10%~85%.

Waterfall plots of the combined model on both the internal and external validation sets were shown in **Figure 6C**. The cutoff value was set by maximizing the Youden index in the derivation set. From the waterfall plots, it can be seen clearly that the combined model can differentiate IAC from AIS/MIA well.

## Discussion

Our research objective was to predict the invasiveness of lung adenocarcinoma manifesting as PSNs with solid components <6 mm. We confirmed that radiomics was superior to the clinical-radiographic model and showed comparable predictive performance to the combined model in differentiating IAC from AIS/MIA. It seems that radiomics can provide a simple and robust prediction method for accurate preoperative judgment of lesion invasiveness.

Our statistical analysis of the patients’ clinical data shows that the incidence of IAC increased with age, male gender, smoking history, and high CEA level, which is consistent with the results of previous studies (32). Although some studies have found that patients with COPD, history of other cancers, and family history of lung cancer were more susceptible to lung cancer (33, 34), our data show that these features have no statistical significance in predicting the invasiveness of PSNs.

Maximum diameter, lesion shape, vacuole sign, and microvascular sign were considered independent predictors for IAC in our study. The size of PSNs is significantly related to the risk of malignancy and is the leading factor in the management of PSNs. In our study, the average maximum diameter of IAC was significantly larger than that of AIS/MIA (1.42 cm vs. 1.09 cm, *p* < 0.001), and the predicted cutoff value for IAC was 1.18 cm. Li et al. (35) and Zhou et al. (36) confirmed that the maximum diameter is an independent predictor of the invasiveness of PSNs, and the predicted cutoff values for IAC in their studies were 1.65 and 1.96 cm, respectively, which were significantly higher than ours. This difference could be due to the fact that other studies included PSNs with solid components ≥6 mm, which may be more aggressive and have larger diameters. As the invasiveness of PSNs increased, their morphology became irregular and the probability of vacuole formation increased due to the proliferation of fibroblasts, tumor cell infiltration in the lung interstitium, and heterogeneity of growth speed inside the tumor (37). Vascular remodeling and sustained angiogenesis play an important role in the early development and progression of tumors (38). When tumor cells infiltrate the pulmonary interstitium, they will create traction on the surrounding blood vessels, causing them to stiffen, twist, and aggregate. Many previous studies have confirmed that irregular shape, vacuole sign, and microvascular sign are more frequently seen in IAC manifesting as PSNs, but no studies considered any of these features as independent predictors (32, 35, 36). The main reason may be that their predictive model was based on the comprehensive analysis of both radiographic features and quantitative parameters instead of only analyzing radiographic features as we did in our study. Zhao et al. (39) found that the mean CT value showed superior predictive performance compared with irregular shape and microvascular sign and was considered to be an independent predictor for IAC. In addition, the different findings could also be due to the exclusion of PSNs with solid components ≥6 mm from our study and the difference in sample size. At present, there are a few studies on PSNs with solid components <6 mm; hence, more studies are needed to determine which radiographic features can be used as the best predictors for invasiveness, and our research results need further verification.

Previous studies have confirmed that measuring the maximum size of solid components or comparing the CTR is more accurate than measuring the maximum diameter of lesions in predicting the invasiveness of PSNs (12, 40). Our study also confirmed that the maximum size of solid components was an independent predictor for IAC. The CTR calculated in our study showed no significant statistical difference between IAC and AIS/MIA. It may be because our study excluded PSNs with solid components ≥6 mm while maintaining the same size enrollment criteria of the lesions (5–30 mm) with previous studies. Although we can clearly identify solid components smaller than 6 mm in PSNs with the aid of automatic segmentation software, errors still exist to a certain extent in the measurement of solid components. No studies focused on the solid component analysis in PSNs with solid components <6 mm before; our research results also need further verification.

In addition to the analysis of radiographic features, there have been many studies using quantitative parameters to predict the invasiveness of PSNs. Lower kurtosis and bigger mass were confirmed as significant differentiators of IAC from AIS/MIA by Chae et al. (41). Ko et al. (27) demonstrated that the total volume and percentage solid volume measurements of PSNs helped differentiate between IAC and AIS/MIA with an accuracy of 73.2%. Therefore, although previous studies are numerous, their results vary due to differences in sample size, quantitative parameters, and analysis methods. For this reason, it is still unclear which parameters contribute the most to the prediction of invasiveness of PSNs. The classification of PSNs can still be challenging for radiologists. However, there were few pieces of research that focused on PSNs with solid component <6 mm. Qi et al. (42) illustrated that the mass of PSNs with solid component <6 mm, with the best cutoff value of 283.2 mg, was the only independent predictor for IAC, and the Az value was 0.859; sensitivity was 68.7% and specificity was 92.9%, which were lower than those achieved by the radiomics model in the external validation set of our study (Az value 0.924, sensitivity 83.5%, specificity 84.2%).

During the growth and evolution of PSNs, as the invasive components containing structural deformities of the stromal elastic fiber framework increase within a homogeneous lepidic or acinar background, the diameter and density of the lesion will increase, the shape will become irregular, and the pixel values will become inhomogeneous. In the seven selected most identifiable radiomics features in our study, gldm_DependenceEntropy and glcm_JointEntropy reflect the uniformity of texture and gray scale, higher Entropy value represents the inhomogeneous of pixels in the tumor and greater probability of IAC. Son et al. (43) reviewed 191 resected ground-glass opacity nodules with little or no solid component and identified entropy as an independent predictor for IAC, which is consistent with our results to some extent. Firstorder_RootMeanSquared and firstorder_90Percentile reflect the brightness and shape_Maximum2DdiameterSlice and shape_Maximum2DdiameterColumn reflect the maximum 2D diameter, which were all positively correlated with the significant density and diameter difference between IAC and AIS/MIA. As the invasiveness of PSNs increase, their shape will become irregular and with higher surface/volume ratio, so it is reasonable that shape_SurfaceVolumeRatio is retained in our radiomics model.

In our study, radiomics outperformed the clinical-radiographic model in predicting the invasiveness of PSNs with solid components <6 mm. In the research of Sun et al. (25), radiomics also showed superior predictive performance compared with the clinical-radiographic model in predicting the invasiveness of NSNs. However, compared with radiomics, our combined model did not demonstrate any significant improvement in predictive ability, which was different from the study of Sun et al. This difference can be explained by the fact that we can only extract finite subjective and semiquantitative information from CT images by the naked eyes, while radiomics can analyze conventional descriptive signs and transform image data into spatial data that can be mined in depth and quantitatively analyzed. Some radiographic features we analyzed are probably included in the features extracted by radiomics. Which surprised us was that both the radiomics and the combined model achieved better results in the external validation set than in the internal validation set in our study. We studied the distribution of contributing features in these datasets, and the distributions of the two most weighted features (gldm_DependenceEntropy, shape_Maximum2DDiameterSlice) were visualized with violin-box plot in **Supplementary Figure S3**. It can be seen that the differences between the distributions of these two features in the positive and negative samples are larger in the external validation set. Therefore, it is understandable that models using these features achieved better results over those of the external validation set.

Deep learning has also been used to predict the invasiveness of PSNs. Kim et al. (44) developed a deep learning model using 2.5D CT images and confirmed that it performed better than the size-based logistic model in distinguishing between IAC and AIS/MIA. However, a deep learning model based on 3D convolutional neural networks in the study by Park et al. (45) showed comparable classification performance with the radiologists’ measurements of solid component size in PSNs. The deep learning method learns features from data and avoids the burden of identifying the effective features manually in images without lesion segmentation. However, deep learning has its own limitations: a large number of cases are needed to train the established model and the interpretability is very limited, users cannot get an effective explanation of the classification results (46, 47). Nevertheless, after expanding the number of cases, we will try to apply the deep learning method to our studies.

Although yearly surveillance CT is recommended for PSNs with solid components <6 mm by Fleischner Society guidelines, the treatment of an individual should also be guided by the probability that the nodule is an IAC and patient preferences. Patients without risk factors (COPD history, family history of lung cancer, etc.) and lower predicted IAC possibility can be followed up routinely and avoid unnecessary surgery. But closer follow-up should be recommended for patients with risk factors and higher predicted IAC possibility. In our study, 16 IAC cases with solid components <6 mm were classified as pT2 stage because of the presence of pleural invasion. So, the accurate IAC prediction in PSNs with solid components <6 mm has important clinical value in the individualized clinical management (routine or closer follow-up, even resection for patients desire surgery) and selection of surgical methods. Our research may provide an individualized clinical management supplement to Fleischner Society guidelines to improve the diagnostic accuracy of radiologists and avoid unnecessary surgery for some patients or provide a clinical basis for some necessary surgeries.

Our study has several limitations. First, all VOIs of the lesion were automatically segmented using software, but as to the lesions with an unclear tumor–lung interface, the segment will be inaccurate and affects the result of data analysis. Manual segmentation is necessary at this time, but the process is tedious and vulnerable to readers’ subjectivity. Second, the clinical-radiographic model in our study was established by the authors #1, #3, and #9 (with 10, 7, and 16 years’ experience in chest CT imaging, respectively); it cannot represent the best diagnostic performance of all radiologists. More radiologists should be enrolled and the “reader performance study” can be performed in our future study. Lastly, numerous radiomics investigations have been published, but the robustness and generalizability of radiomics models still remain questionable and need to be verified by clinical practice. Although we have confirmed that the radiomics features we extracted achieved satisfactory inter- and intra-observer reproducibility and an external validation set from another hospital was also introduced in our study, multicenter research is still necessary in the future study.

## Conclusions

Radiomics has been proven to achieve outstanding classification performance in classifying PSNs with solid components <6 mm. It has the potential as an independent diagnostic tool to improve the classification ability of radiologists or thoracic surgeons and save their time and effort without compromising diagnostic accuracy.

## Data availability statement

The raw data supporting the conclusions of this article will be made available by the authors, without undue reservation.

## Ethics statement

This study was reviewed and approved by the ethical committee of the first affiliated hospital of Nanjing medical university approved this retrospective study and waived the informed consent for the patients (approval number: 2021-SR-053). Written informed consent for participation was not required for this study in accordance with the national legislation and the institutional requirements.

## Author contributions

TZ, CZ and MY: conceptualization and methodology. TZ, CZ and GY: data curation and formal analysis. MY and QZ: funding acquisition, project administration and supervision. TZ, CZ, YZ, YS and MY: investigation. TZ, CZ, HW and GY: software. TZ, CZ, HL, MY and GY: validation. TZ and CZ: writing-original draft. MY and GY: writing-review and editing. All authors contributed to the article and approved the submitted version.

## Funding

This work was supported by grants from the National Nature Science Foundation of China (81801693).

## Conflict of interest

The authors declare that the research was conducted in the absence of any commercial or financial relationships that could be construed as a potential conflict of interest.

## Publisher’s note

All claims expressed in this article are solely those of the authors and do not necessarily represent those of their affiliated organizations, or those of the publisher, the editors and the reviewers. Any product that may be evaluated in this article, or claim that may be made by its manufacturer, is not guaranteed or endorsed by the publisher.
