# Supplementary material for: A radiomics nomogram for invasiveness prediction in lung adenocarcinoma manifesting as part-solid nodules with solid components smaller than 6 mm
Source: Front Oncol. 2022 Aug 11;12:900049. doi: 10.3389/fonc.2022.900049 (PMC9406823; doi:10.3389/fonc.2022.900049)
Supplement: Supplementary file 1 [file DataSheet_1.docx]

Supplementary Material

**Supplementary Table 1:**  CT scanning parameters and number of patients performed by each scanner.

| **Setting** | **SOMATOM Definition AS+** | **SOMATOM Sensation 16** | **GE Discovery CT750 HD** | **GE LightSpeed VCT** |
| --- | --- | --- | --- | --- |
| Tube voltage | 120 kVp | 120 kVp | 120 kVp | 120 kVp |
| Tube current | 110 mAs | 110 mAs | 200 mAs | 200 mAs |
| Pitch | 1.0 | 0.8 | 0.984:1 | 0.984:1 |
| Collimation | 0.6mm*64 | 0.75mm*16 | 0.625mm*64 | 0.625mm*64 |
| Rotation time | 0.33 s/rot | 0.35 s/rot | 0.5 s/rot | 0.5 s/rot |
| SFOV | 50cm | 50cm | 50cm | 50cm |
| Slice thickness of reconstruction | 1.5mm | 1.5mm | 1.25mm | 1.25mm |
| Slice interval of reconstruction | 1.5mm | 1.5mm | 1.25mm | 1.25mm |
| Reconstruction algorithm | Medium sharp | Medium sharp | STND | STND |
| Number of patients | 616 | 118 | 278 | 198 |
| Scanning methods | Plain scan | Plain scan | Plain scan | Plain scan |

**Supplementary Table 2:** Inter-observer agreement for assessing CT radiographic characteristics

| **Characteristics** | **ICCs ( 95%CI )** | **Kappa values ( 95%CI )** |
| --- | --- | --- |
| Maximum axial diameter of the lesion | 0.83 (0.67-0.99) | – |
| Maximum axial diameter of the solid component | 0.65 (0.34-0.96) | – |
| CTR | 0.70 (0.35-1.05) |  |
| Lesion location | – | 1.00 |
| Lesion shape | – | 0.73 (0.50-0.95) |
| Lesion border | – | 0.83 (0.61-1.05) |
| Vacuole sign | – | 0.79 (0.54-1.04) |
| Air bronchogram | – | 0.70 (0.45-0.95) |
| Microvascular sign | – | 0.71 (0.45-0.98) |
| Pleural indentation | – | 0.93 (0.79-1.07) |

ICC, intraclass correlation coefficient; CI, confidence interval; CTR, consolidation-to-tumor ratio.

**Supplementary Table 3:** Inter-observer ICCs of the extracted 105 features

| FeatureName | ICC | 95%CI |
| --- | --- | --- |
| lesion_original_firstorder_10Percentile | 0.978 | [0.96 0.99] |
| lesion_original_firstorder_90Percentile | 0.947 | [0.91 0.97] |
| lesion_original_firstorder_Energy | 0.983 | [0.97 0.99] |
| lesion_original_firstorder_Entropy | 0.99 | [0.98 0.99] |
| lesion_original_firstorder_InterquartileRange | 0.987 | [0.98 0.99] |
| lesion_original_firstorder_Kurtosis | 0.964 | [0.93 0.98] |
| lesion_original_firstorder_Maximum | 0.944 | [0.9 0.97] |
| lesion_original_firstorder_Mean | 0.976 | [0.96 0.99] |
| lesion_original_firstorder_MeanAbsoluteDeviation | 0.971 | [0.95 0.98] |
| lesion_original_firstorder_Median | 0.978 | [0.96 0.99] |
| lesion_original_firstorder_Minimum | 0.984 | [0.97 0.99] |
| lesion_original_firstorder_Range | 0.947 | [0.9 0.97] |
| lesion_original_firstorder_RobustMeanAbsoluteDeviation | 0.979 | [0.96 0.99] |
| lesion_original_firstorder_RootMeanSquared | 0.962 | [0.94 0.98] |
| lesion_original_firstorder_Skewness | 0.954 | [0.92 0.97] |
| lesion_original_firstorder_TotalEnergy | 0.984 | [0.97 0.99] |
| lesion_original_firstorder_Uniformity | 0.996 | [0.99 1. ] |
| lesion_original_firstorder_Variance | 0.936 | [0.9 0.96] |
| lesion_original_glcm_Autocorrelation | 0.923 | [0.87 0.95] |
| lesion_original_glcm_ClusterProminence | 0.802 | [0.69 0.88] |
| lesion_original_glcm_ClusterShade | 0.832 | [0.73 0.9 ] |
| lesion_original_glcm_ClusterTendency | 0.892 | [0.83 0.93] |
| lesion_original_glcm_Contrast | 0.966 | [0.94 0.98] |
| lesion_original_glcm_Correlation | 0.942 | [0.9 0.96] |
| lesion_original_glcm_DifferenceAverage | 0.984 | [0.97 0.99] |
| lesion_original_glcm_DifferenceEntropy | 0.991 | [0.98 0.99] |
| lesion_original_glcm_DifferenceVariance | 0.945 | [0.91 0.97] |
| lesion_original_glcm_Id | 0.996 | [0.99 1. ] |
| lesion_original_glcm_Idm | 0.997 | [1. 1.] |
| lesion_original_glcm_Idmn | 0.97 | [0.95 0.98] |
| lesion_original_glcm_Idn | 0.967 | [0.94 0.98] |
| lesion_original_glcm_Imc1 | 0.978 | [0.96 0.99] |
| lesion_original_glcm_Imc2 | 0.993 | [0.99 1. ] |
| lesion_original_glcm_InverseVariance | 0.997 | [1. 1.] |
| lesion_original_glcm_JointAverage | 0.953 | [0.92 0.97] |
| lesion_original_glcm_JointEnergy | 0.994 | [0.99 1. ] |
| lesion_original_glcm_JointEntropy | 0.985 | [0.98 0.99] |
| lesion_original_glcm_MaximumProbability | 0.993 | [0.99 1. ] |
| lesion_original_glcm_SumEntropy | 0.984 | [0.97 0.99] |
| lesion_original_glcm_SumSquares | 0.917 | [0.86 0.95] |
| lesion_original_gldm_DependenceEntropy | 0.976 | [0.96 0.99] |
| lesion_original_gldm_DependenceNonUniformity | 0.978 | [0.96 0.99] |
| lesion_original_gldm_DependenceNonUniformityNormalized | 0.98 | [0.97 0.99] |
| lesion_original_gldm_DependenceVariance | 0.998 | [1. 1.] |
| lesion_original_gldm_GrayLevelNonUniformity | 0.989 | [0.98 0.99] |
| lesion_original_gldm_GrayLevelVariance | 0.936 | [0.9 0.96] |
| lesion_original_gldm_HighGrayLevelEmphasis | 0.933 | [0.89 0.96] |
| lesion_original_gldm_LargeDependenceEmphasis | 0.998 | [1. 1.] |
| lesion_original_gldm_LargeDependenceHighGrayLevelEmphasis | 0.93 | [0.89 0.96] |
| lesion_original_gldm_LargeDependenceLowGrayLevelEmphasis | 0.983 | [0.97 0.99] |
| lesion_original_gldm_LowGrayLevelEmphasis | 0.91 | [0.85 0.95] |
| lesion_original_gldm_SmallDependenceEmphasis | 0.986 | [0.98 0.99] |
| lesion_original_gldm_SmallDependenceHighGrayLevelEmphasis | 0.935 | [0.89 0.96] |
| lesion_original_gldm_SmallDependenceLowGrayLevelEmphasis | 0.861 | [0.78 0.91] |
| lesion_original_glrlm_GrayLevelNonUniformity | 0.987 | [0.98 0.99] |
| lesion_original_glrlm_GrayLevelNonUniformityNormalized | 0.996 | [0.99 1. ] |
| lesion_original_glrlm_GrayLevelVariance | 0.936 | [0.9 0.96] |
| lesion_original_glrlm_HighGrayLevelRunEmphasis | 0.933 | [0.89 0.96] |
| lesion_original_glrlm_LongRunEmphasis | 0.997 | [1. 1.] |
| lesion_original_glrlm_LongRunHighGrayLevelEmphasis | 0.932 | [0.89 0.96] |
| lesion_original_glrlm_LongRunLowGrayLevelEmphasis | 0.922 | [0.87 0.95] |
| lesion_original_glrlm_LowGrayLevelRunEmphasis | 0.908 | [0.85 0.94] |
| lesion_original_glrlm_RunEntropy | 0.988 | [0.98 0.99] |
| lesion_original_glrlm_RunLengthNonUniformity | 0.975 | [0.96 0.99] |
| lesion_original_glrlm_RunLengthNonUniformityNormalized | 0.995 | [0.99 1. ] |
| lesion_original_glrlm_RunPercentage | 0.996 | [0.99 1. ] |
| lesion_original_glrlm_RunVariance | 0.998 | [1. 1.] |
| lesion_original_glrlm_ShortRunEmphasis | 0.996 | [0.99 1. ] |
| lesion_original_glrlm_ShortRunHighGrayLevelEmphasis | 0.933 | [0.89 0.96] |
| lesion_original_glrlm_ShortRunLowGrayLevelEmphasis | 0.906 | [0.85 0.94] |
| lesion_original_glszm_GrayLevelNonUniformity | 0.979 | [0.96 0.99] |
| lesion_original_glszm_GrayLevelNonUniformityNormalized | 0.994 | [0.99 1. ] |
| lesion_original_glszm_GrayLevelVariance | 0.935 | [0.89 0.96] |
| lesion_original_glszm_HighGrayLevelZoneEmphasis | 0.933 | [0.89 0.96] |
| lesion_original_glszm_LargeAreaEmphasis | 0.996 | [0.99 1. ] |
| lesion_original_glszm_LargeAreaHighGrayLevelEmphasis | 0.995 | [0.99 1. ] |
| lesion_original_glszm_LargeAreaLowGrayLevelEmphasis | 0.995 | [0.99 1. ] |
| lesion_original_glszm_LowGrayLevelZoneEmphasis | 0.917 | [0.86 0.95] |
| lesion_original_glszm_SizeZoneNonUniformity | 0.982 | [0.97 0.99] |
| lesion_original_glszm_SizeZoneNonUniformityNormalized | 0.972 | [0.95 0.98] |
| lesion_original_glszm_SmallAreaEmphasis | 0.973 | [0.96 0.98] |
| lesion_original_glszm_SmallAreaHighGrayLevelEmphasis | 0.934 | [0.89 0.96] |
| lesion_original_glszm_SmallAreaLowGrayLevelEmphasis | 0.87 | [0.79 0.92] |
| lesion_original_glszm_ZoneEntropy | 0.977 | [0.96 0.99] |
| lesion_original_glszm_ZonePercentage | 0.99 | [0.98 0.99] |
| lesion_original_glszm_ZoneVariance | 0.996 | [0.99 1. ] |
| lesion_original_ngtdm_Busyness | 0.987 | [0.98 0.99] |
| lesion_original_ngtdm_Coarseness | 0.977 | [0.96 0.99] |
| lesion_original_ngtdm_Complexity | 0.943 | [0.89 0.97] |
| lesion_original_ngtdm_Contrast | 0.985 | [0.98 0.99] |
| lesion_original_ngtdm_Strength | 0.909 | [0.85 0.94] |
| lesion_original_shape_Elongation | 0.86 | [0.78 0.91] |
| lesion_original_shape_Flatness | 0.944 | [0.91 0.97] |
| lesion_original_shape_LeastAxisLength | 0.976 | [0.96 0.99] |
| lesion_original_shape_MajorAxisLength | 0.992 | [0.99 1. ] |
| lesion_original_shape_Maximum2DDiameterColumn | 0.98 | [0.97 0.99] |
| lesion_original_shape_Maximum2DDiameterRow | 0.973 | [0.96 0.98] |
| lesion_original_shape_Maximum2DDiameterSlice | 0.97 | [0.95 0.98] |
| lesion_original_shape_Maximum3DDiameter | 0.984 | [0.97 0.99] |
| lesion_original_shape_MeshVolume | 0.977 | [0.96 0.99] |
| lesion_original_shape_MinorAxisLength | 0.966 | [0.94 0.98] |
| lesion_original_shape_Sphericity | 0.887 | [0.81 0.93] |
| lesion_original_shape_SurfaceArea | 0.987 | [0.98 0.99] |
| lesion_original_shape_SurfaceVolumeRatio | 0.947 | [0.91 0.97] |
| lesion_original_shape_VoxelVolume | 0.977 | [0.96 0.99] |

ICC, intraclass correlation coefficient; CI, confidence interval.

**Supplementary Table 4:** Intra-observer ICCs of the extracted 105 features

| FeatureName | ICC | 95%CI |
| --- | --- | --- |
| lesion_original_firstorder_10Percentile | 0.994 | [0.99 1. ] |
| lesion_original_firstorder_90Percentile | 0.979 | [0.96 0.99] |
| lesion_original_firstorder_Energy | 0.999 | [1. 1.] |
| lesion_original_firstorder_Entropy | 0.996 | [0.99 1. ] |
| lesion_original_firstorder_InterquartileRange | 0.995 | [0.99 1. ] |
| lesion_original_firstorder_Kurtosis | 0.983 | [0.97 0.99] |
| lesion_original_firstorder_Maximum | 0.965 | [0.94 0.98] |
| lesion_original_firstorder_Mean | 0.994 | [0.98 1. ] |
| lesion_original_firstorder_MeanAbsoluteDeviation | 0.988 | [0.98 0.99] |
| lesion_original_firstorder_Median | 0.995 | [0.99 1. ] |
| lesion_original_firstorder_Minimum | 0.997 | [0.99 1. ] |
| lesion_original_firstorder_Range | 0.972 | [0.95 0.98] |
| lesion_original_firstorder_RobustMeanAbsoluteDeviation | 0.991 | [0.99 0.99] |
| lesion_original_firstorder_RootMeanSquared | 0.985 | [0.97 0.99] |
| lesion_original_firstorder_Skewness | 0.977 | [0.96 0.99] |
| lesion_original_firstorder_TotalEnergy | 0.998 | [1. 1.] |
| lesion_original_firstorder_Uniformity | 0.999 | [1. 1.] |
| lesion_original_firstorder_Variance | 0.969 | [0.95 0.98] |
| lesion_original_glcm_Autocorrelation | 0.984 | [0.97 0.99] |
| lesion_original_glcm_ClusterProminence | 0.906 | [0.84 0.94] |
| lesion_original_glcm_ClusterShade | 0.925 | [0.87 0.96] |
| lesion_original_glcm_ClusterTendency | 0.942 | [0.9 0.97] |
| lesion_original_glcm_Contrast | 0.977 | [0.96 0.99] |
| lesion_original_glcm_Correlation | 0.975 | [0.96 0.99] |
| lesion_original_glcm_DifferenceAverage | 0.991 | [0.98 1. ] |
| lesion_original_glcm_DifferenceEntropy | 0.995 | [0.99 1. ] |
| lesion_original_glcm_DifferenceVariance | 0.958 | [0.92 0.98] |
| lesion_original_glcm_Id | 0.999 | [1. 1.] |
| lesion_original_glcm_Idm | 0.999 | [1. 1.] |
| lesion_original_glcm_Idmn | 0.981 | [0.97 0.99] |
| lesion_original_glcm_Idn | 0.985 | [0.98 0.99] |
| lesion_original_glcm_Imc1 | 0.998 | [1. 1.] |
| lesion_original_glcm_Imc2 | 0.999 | [1. 1.] |
| lesion_original_glcm_InverseVariance | 0.999 | [1. 1.] |
| lesion_original_glcm_JointAverage | 0.99 | [0.98 0.99] |
| lesion_original_glcm_JointEnergy | 0.998 | [1. 1.] |
| lesion_original_glcm_JointEntropy | 0.997 | [1. 1.] |
| lesion_original_glcm_MaximumProbability | 0.998 | [1. 1.] |
| lesion_original_glcm_SumEntropy | 0.992 | [0.99 1. ] |
| lesion_original_glcm_SumSquares | 0.954 | [0.92 0.97] |
| lesion_original_gldm_DependenceEntropy | 0.993 | [0.99 1. ] |
| lesion_original_gldm_DependenceNonUniformity | 0.999 | [1. 1.] |
| lesion_original_gldm_DependenceNonUniformityNormalized | 0.995 | [0.99 1. ] |
| lesion_original_gldm_DependenceVariance | 0.923 | [0.87 0.95] |
| lesion_original_gldm_GrayLevelNonUniformity | 0.962 | [0.95 0.97] |
| lesion_original_gldm_GrayLevelVariance | 0.969 | [0.95 0.98] |
| lesion_original_gldm_HighGrayLevelEmphasis | 0.987 | [0.97 0.99] |
| lesion_original_gldm_LargeDependenceEmphasis | 0.882 | [0.81 0.93] |
| lesion_original_gldm_LargeDependenceHighGrayLevelEmphasis | 0.988 | [0.98 0.99] |
| lesion_original_gldm_LargeDependenceLowGrayLevelEmphasis | 0.999 | [1. 1.] |
| lesion_original_gldm_LowGrayLevelEmphasis | 0.977 | [0.96 0.99] |
| lesion_original_gldm_SmallDependenceEmphasis | 0.996 | [0.99 1. ] |
| lesion_original_gldm_SmallDependenceHighGrayLevelEmphasis | 0.986 | [0.97 0.99] |
| lesion_original_gldm_SmallDependenceLowGrayLevelEmphasis | 0.96 | [0.93 0.98] |
| lesion_original_glrlm_GrayLevelNonUniformity | 0.892 | [0.82 0.94] |
| lesion_original_glrlm_GrayLevelNonUniformityNormalized | 0.999 | [1. 1.] |
| lesion_original_glrlm_GrayLevelVariance | 0.969 | [0.95 0.98] |
| lesion_original_glrlm_HighGrayLevelRunEmphasis | 0.987 | [0.97 0.99] |
| lesion_original_glrlm_LongRunEmphasis | 0.999 | [1. 1.] |
| lesion_original_glrlm_LongRunHighGrayLevelEmphasis | 0.987 | [0.97 0.99] |
| lesion_original_glrlm_LongRunLowGrayLevelEmphasis | 0.982 | [0.97 0.99] |
| lesion_original_glrlm_LowGrayLevelRunEmphasis | 0.977 | [0.96 0.99] |
| lesion_original_glrlm_RunEntropy | 0.995 | [0.99 1. ] |
| lesion_original_glrlm_RunLengthNonUniformity | 0.973 | [0.94 0.98] |
| lesion_original_glrlm_RunLengthNonUniformityNormalized | 0.999 | [1. 1.] |
| lesion_original_glrlm_RunPercentage | 0.999 | [1. 1.] |
| lesion_original_glrlm_RunVariance | 0.953 | [0.92 0.97] |
| lesion_original_glrlm_ShortRunEmphasis | 0.999 | [1. 1.] |
| lesion_original_glrlm_ShortRunHighGrayLevelEmphasis | 0.987 | [0.97 0.99] |
| lesion_original_glrlm_ShortRunLowGrayLevelEmphasis | 0.975 | [0.96 0.99] |
| lesion_original_glszm_GrayLevelNonUniformity | 0.999 | [1. 1.] |
| lesion_original_glszm_GrayLevelNonUniformityNormalized | 0.998 | [1. 1.] |
| lesion_original_glszm_GrayLevelVariance | 0.964 | [0.94 0.98] |
| lesion_original_glszm_HighGrayLevelZoneEmphasis | 0.985 | [0.97 0.99] |
| lesion_original_glszm_LargeAreaEmphasis | 0.932 | [0.88 0.95] |
| lesion_original_glszm_LargeAreaHighGrayLevelEmphasis | 0.835 | [0.72 0.91 ] |
| lesion_original_glszm_LargeAreaLowGrayLevelEmphasis | 0.966 | [0.94 0.98] |
| lesion_original_glszm_LowGrayLevelZoneEmphasis | 0.978 | [0.96 0.99] |
| lesion_original_glszm_SizeZoneNonUniformity | 0.999 | [1. 1.] |
| lesion_original_glszm_SizeZoneNonUniformityNormalized | 0.991 | [0.99 0.99] |
| lesion_original_glszm_SmallAreaEmphasis | 0.992 | [0.99 1. ] |
| lesion_original_glszm_SmallAreaHighGrayLevelEmphasis | 0.984 | [0.97 0.99] |
| lesion_original_glszm_SmallAreaLowGrayLevelEmphasis | 0.968 | [0.95 0.98] |
| lesion_original_glszm_ZoneEntropy | 0.991 | [0.98 0.99] |
| lesion_original_glszm_ZonePercentage | 0.998 | [1. 1.] |
| lesion_original_glszm_ZoneVariance | 0.892 | [0.83 0.93] |
| lesion_original_ngtdm_Busyness | 0.994 | [0.99 1. ] |
| lesion_original_ngtdm_Coarseness | 0.997 | [1. 1.] |
| lesion_original_ngtdm_Complexity | 0.967 | [0.94 0.98] |
| lesion_original_ngtdm_Contrast | 0.994 | [0.99 1. ] |
| lesion_original_ngtdm_Strength | 0.961 | [0.93 0.98] |
| lesion_original_shape_Elongation | 0.968 | [0.95 0.98] |
| lesion_original_shape_Flatness | 0.975 | [0.96 0.98] |
| lesion_original_shape_LeastAxisLength | 0.998 | [1. 1.] |
| lesion_original_shape_MajorAxisLength | 0.998 | [1. 1.] |
| lesion_original_shape_Maximum2DDiameterColumn | 0.997 | [0.99 1. ] |
| lesion_original_shape_Maximum2DDiameterRow | 0.992 | [0.98 1. ] |
| lesion_original_shape_Maximum2DDiameterSlice | 0.99 | [0.98 0.99] |
| lesion_original_shape_Maximum3DDiameter | 0.994 | [0.99 1. ] |
| lesion_original_shape_MeshVolume | 0.999 | [1. 1.] |
| lesion_original_shape_MinorAxisLength | 0.997 | [0.99 1. ] |
| lesion_original_shape_Sphericity | 0.908 | [0.5 0.97] |
| lesion_original_shape_SurfaceArea | 0.998 | [0.98 1. ] |
| lesion_original_shape_SurfaceVolumeRatio | 0.972 | [0.92 0.99] |
| lesion_original_shape_VoxelVolume | 0.999 | [1. 1.] |

ICC, intraclass correlation coefficient; CI, confidence interval.

**Supplementary Figure 1** The identification and measurement of the solid components in PSNs in our study. (A) CT threshold of the solid components in PSNs was set to > -188 HU referring to previous studies. 3D Slicer software can automatically identify the solid components in PSNs from the axial, coronal and sagittal images. (B, C, D) According to the automatic segment results of the solid components in 3D Slicer software (arrow), authors #1 and #3 separately measured the maximum diameter of the solid components from the axial, coronal and sagittal images in PACS. PSNs with solid components ≥ 6 mm were excluded from the study.

**Supplementary Figure 2** The volume of interest (VOI) segmentation of the lesion. (A) The original CT image of the lesion. (B) VOI was automatically segmented using a homemade software MultiLabel (version 1.1, Shanghai Key laboratory of Magnetic Resonance, East China Normal University, China). Manually adjustment for precise edge of VOI was performed by author #1 if the border of lesion was undefined or to ensure the large vessels and bronchioles were excluded from VOI. (C) The 3D image of the segmented lesion.

**Supplementary Figure 3** Distributions of two most important contributing features in the radiomics model, in the positive and negative samples in three different datasets.
