# Supplementary figures and images for: A radiomics nomogram for invasiveness prediction in lung adenocarcinoma manifesting as part-solid nodules with solid components smaller than 6 mm
Source: Front Oncol. 2022 Aug 11;12:900049. doi: 10.3389/fonc.2022.900049 (PMC9406823; doi:10.3389/fonc.2022.900049)

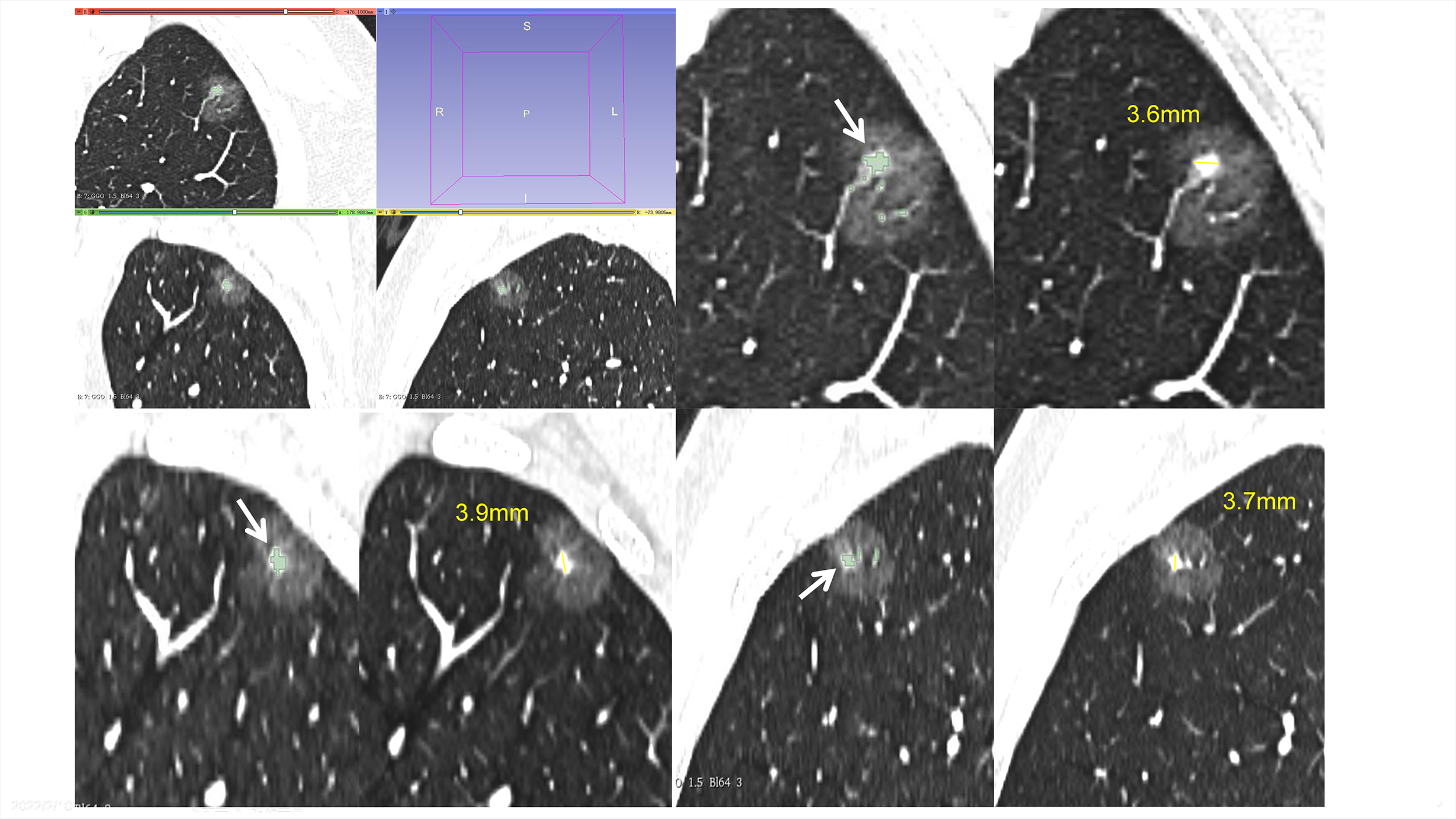

Supplement: Supplementary file 2 [file Image_1.tif]

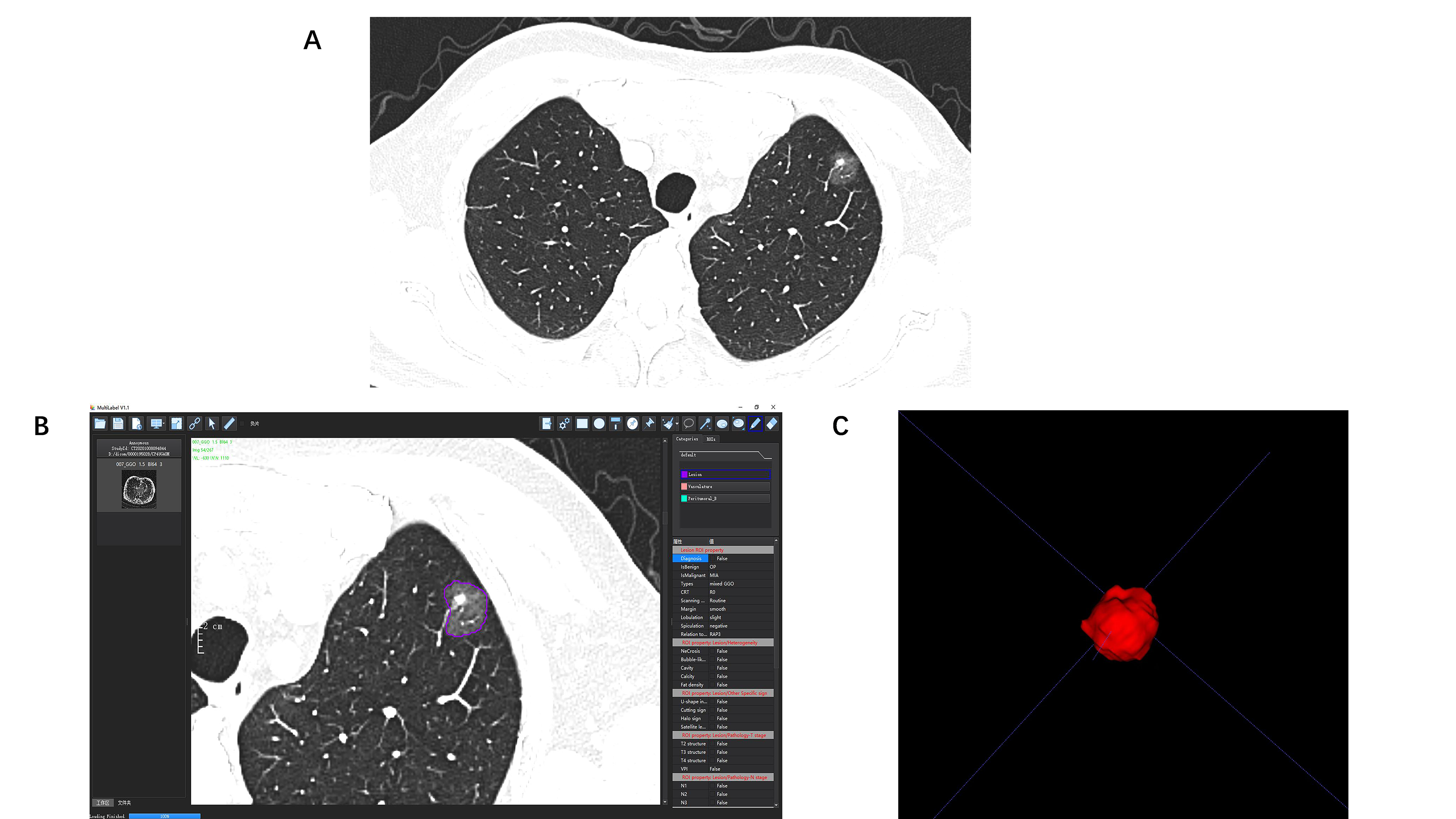

Supplement: Supplementary file 3 [file Image_2.tif]

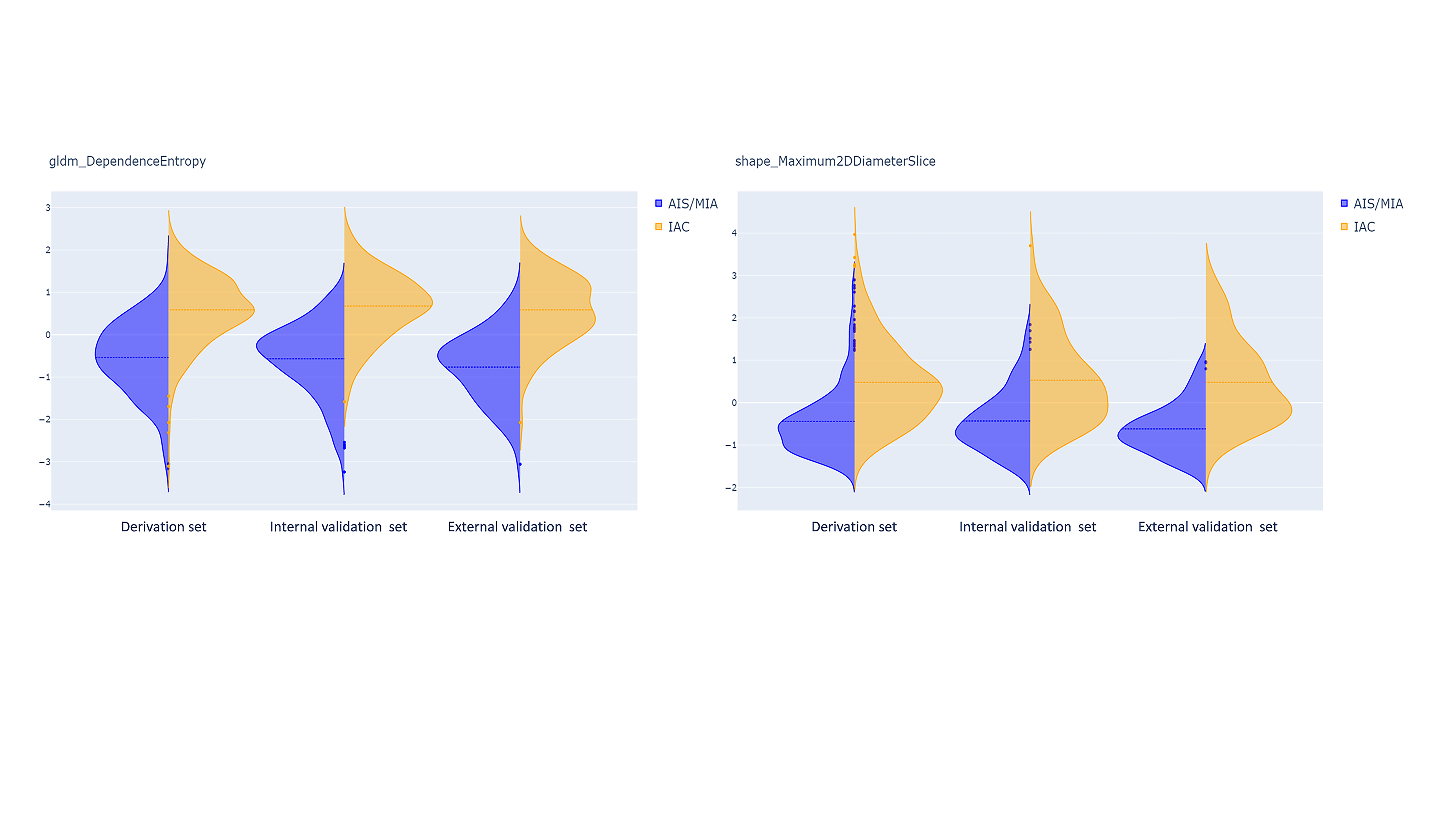

Supplement: Supplementary file 4 [file Image_3.tif]
